# Differentiation potential of STRO-1^+ ^dental pulp stem cells changes during cell passaging

**DOI:** 10.1186/1471-2121-11-32

**Published:** 2010-05-08

**Authors:** Jinhua Yu, Huixia He, Chunbo Tang, Guangdong Zhang, Yuanfei Li, Ruoning Wang, Junnan Shi, Yan Jin

**Affiliations:** 1 Institute of Stomatology, Nanjing Medical University, Nanjing, Jiangsu 210029, China; 2 Institute of Dental Research of Chinese PLA, General Hospital and Postgraduate Military Medical School, Beijing 100853, China; 3 State Key Laboratory of Cancer Biology, Fourth Military Medical University, Xi'an, Shaanxi 710032, China; 4 Department of Experimental Therapeutics, The University of Texas MD Anderson Cancer Center, 1515 Holcombe Blvd, Houston, TX 77030, USA; 5 School of Stomatology, Fourth Military Medical University, Xi'an, Shaanxi 710032, China

## Abstract

**Background:**

Dental pulp stem cells (DPSCs) can be driven into odontoblast, osteoblast, and chondrocyte lineages in different inductive media. However, the differentiation potential of naive DPSCs after serial passaging in the routine culture system has not been fully elucidated.

**Results:**

DPSCs were isolated from human/rat dental pulps by the magnetic activated cell sorting based on STRO-1 expression, cultured and passaged in the conventional culture media. The biological features of STRO-1^+ ^DPSCs at the 1^st ^and 9^th ^passages were investigated. During the long-term passage, the proliferation ability of human STRO-1^+ ^DPSCs was downregulated as indicated by the growth kinetics. When compared with STRO-1^+ ^DPSCs at the 1^st ^passage (DPSC-P1), the expression of mature osteoblast-specific genes/proteins (alkaline phosphatase, bone sialoprotein, osterix, and osteopontin), odontoblast-specific gene/protein (dentin sialophosphoprotein and dentin sialoprotein), and chondrocyte-specific gene/protein (type II collagen) was significantly upregulated in human STRO-1^+ ^DPSCs at the 9^th ^passage (DPSC-P9). Furthermore, human DPSC-P9 cells in the mineralization-inducing media presented higher levels of alkaline phosphatase at day 3 and day 7 respectively, and produced more mineralized matrix than DPSC-P9 cells at day 14. *In vivo *transplantation results showed that rat DPSC-P1 cell pellets developed into dentin, bone and cartilage structures respectively, while DPSC-P9 cells can only generate bone tissues.

**Conclusions:**

These findings suggest that STRO-1^+ ^DPSCs consist of several interrelated subpopulations which can spontaneously differentiate into odontoblasts, osteoblasts, and chondrocytes. The differentiation capacity of these DPSCs changes during cell passaging, and DPSCs at the 9^th ^passage restrict their differentiation potential to the osteoblast lineage *in vivo*.

## Background

During the odontogenesis, reciprocal epithelial-mesenchymal interactions are of paramount importance to the tooth initiation and subsequent dental morphogenesis [1-4]. At the beginning of odontogenesis, the ectomesenchyme provide the initial inductive signals and bring about the formation of dental placode. Subsequent cell proliferation, condensation, polarization, and differentiation of the epithelium and mesenchyme contribute to the crown morphogenesis [3,5]. At the cap stage, dental mesenchyme has acquired the dentinogenic capacity to instruct subsequent tooth development [6]. When the first layer of dentin matrix is generated between dental epithelium and mesenchyme, the reciprocal interactions are interrupted because of the dentin barrier. Dental mesenchymal cells in this stage can still perform the regular dentinogenesis without the epithelial-mesenchymal interactions [7]. At the following developmental stages, the primary and secondary dentinogenesis mediated by the dental mesenchyme are still going on around the dental pulp in the absence of epithelial components throughout the rest life of a healthy tooth. Our previous study has proved that induced dental pulp stem cells (DPSCs) can undergo the odontoblastic differentiation and dentinogenesis in the presence of epithelial signals [8], however, whether naive DPSCs can spontaneously give birth to the odontoblasts and solely carry out the dentinogenesis in the absence of inductive agents (e.g. epithelial signals and extracellular matrix) has not been fully elucidated.

Many studies have demonstrated that DPSCs have the ability to undertake the self renewal and differentiate into the neurogenic, osteogenic, dentinogenic, and myogenic cell lineages in different inductive media [9,10]. As a type of adult stem cells, DPSCs usually perform the asymmetric cell division, which gives rise to one daughter cell with stem-cell fate and another which can pursue further cell divisions to generate differentiated progenies. For the lack of specific cell surface markers, the identification of DPSCs mainly relies on their biological features, including small cell volume, high proliferation potency, high clonogenicity, self-renewal, and multiple differentiation potential [9,10]. DPSCs *in vivo *usually remain quiescent within adult dental pulps, but respond during injury to produce progenies with high proliferative potential which can differentiate into terminally differentiated odontoblasts. Thus, the amount of DPSCs in the normal dental pulp remains relatively constant. When one DPSC divide 9 times, it will generate 9 lineage-specific progeny cells plus one unaltered daughter stem cell. These lineage-specific progenies *in vivo *may bring about different cell types which contribute to the maintenance and homeostasis of dental pulp tissues. However, little is known about the fate of these lineage-specific progenies after serial passaging.

To date, there is no optimal culture medium that can allow adult stem cell amplification without differentiation [11,12], it is reasonable that naive DPSCs *in vitro *can spontaneously differentiate (termed self-differentiation) into mature cell lineages via asymmetric cell division. For this purpose, this study was designed to evaluate the differentiation potential of DPSCs at different passages in the routine culture system. The findings presented in this study indicate that STRO-1^+ ^DPSCs *in vitro *can spontaneously differentiate into several mature cell lineages with passage time, while the differentiation capacity of these stem cells changes during cell passaging and DPSCs at the 9^th ^passage *in vivo *restrict their differentiation potential to osteoblast lineages.

## Results

### Morphological and growth features of STRO-1^+ ^DPSCs at the 1^st ^and 9^th ^passages

**Figure 1:**
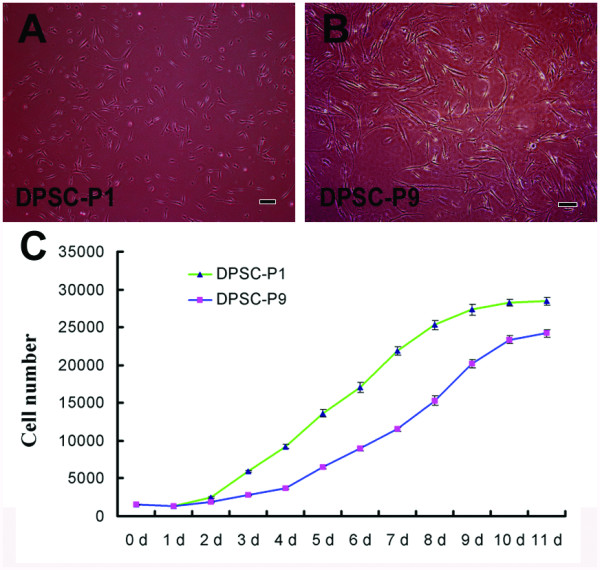
**Morphological appearance and growth curve of DPSCs at different passages**. (**A**) DPSCs at the 1^st ^passage. (**B**) DPSCs at the 9^th ^passage. Many DPSCs became enlarged and elongated with one or two cellular processes. (**C**) Growth curve of DPSCs at the 1^st ^and 9^th ^passages. Population doubling time (PDT) in DPSC-P1 and DPSC-P9 group was 1.83 d and 3.42 d respectively. Values are mean ± s.d., n = 3. Scale bars: 50 μm.

To investigate the proliferation features of STRO-1^+ ^DPSCs at different passages, growth curve was plotted with culture time and population doubling time (PDT) was calculated according to the Patterson formulation. As compared with polymorphic DPSCs (Figure 1A) at the 1^st ^passage (DPSC-P1), some DPSCs at the 9^th ^passage (DPSC-P9) displayed the enlarged cell bodies and elongated appearance with one or two long cellular processes (Figure 1B). As showed in the growth curve (Figure 1C), cells began to grow exponentially at day 2 in DPSC-P1 group and at day 4 in DPSC-P9 group respectively after an initial lag phase. In the logarithmic phase, population doubling time (PDT) in DPSC-P9 group (3.42 d) was longer than that in DPSC-P1 (1.83 d) group, indicating a downregulated proliferation potential in DPSC-P9 cells.

### Gene and protein expression of STRO-1^+ ^DPSCs at the 1^st ^and 9^th ^passages

**Figure 2:**
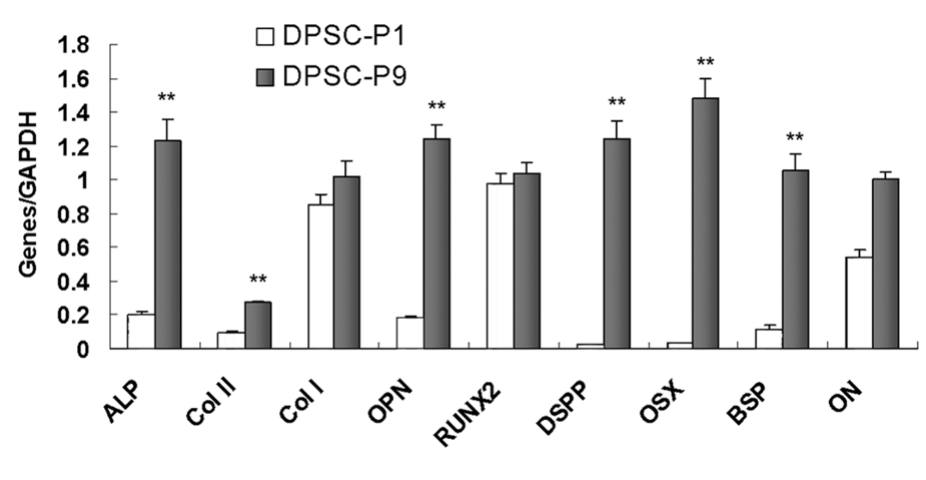
**Gene expression of DPSCs at different passages by realtime reverse transcriptase-polymerase chain reaction**. GAPDH was used as an internal control. Values are mean ± s.d., n = 6 (**P < 0.001).

**Figure 3:**
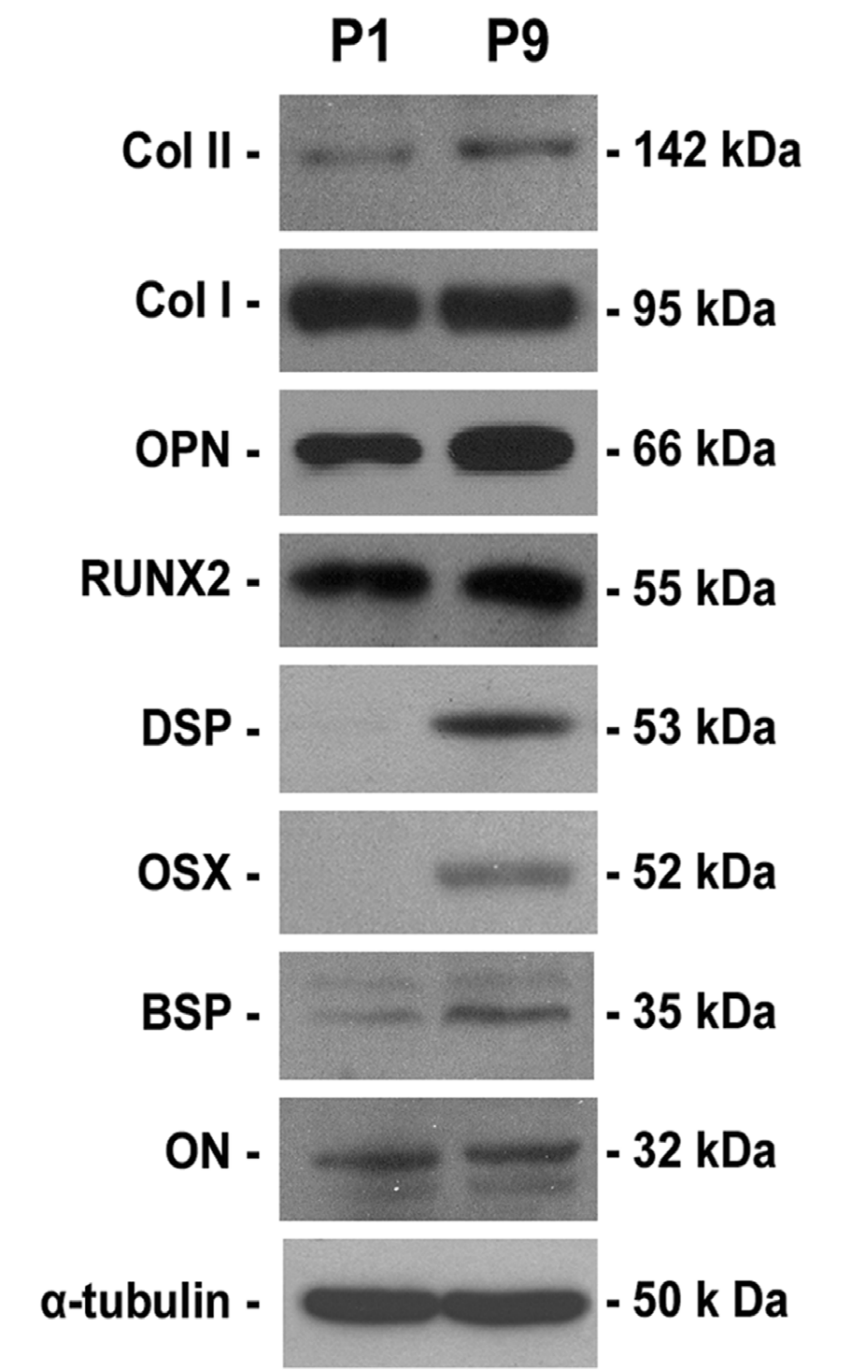
**Protein expression of DPSCs at different passages by western blot analyses**. α-tubulin was used as a control.

To evaluate the phenotype changes of *in vitro *DPSCs during the long-term passage, the gene/protein expression of STRO-1^+ ^DPSCs at the 1^st ^passage and 9^th ^passage was detected by means of real-time RT-PCR and western blot respectively. The gene expression of alkaline phosphatase (*ALP*, mature osteo-/odontoblast marker), osteopontin (*OPN*, mature osteoblast marker), osterix (*OSX*, mature osteoblast marker), bone sialoprotein (*BSP*, mature osteoblast marker), dentin sialophosphoprotein (*DSPP*, odontoblast marker), and type II collagen (*COL *II, chondrocyte marker) was significantly upregulated in DPSC-P9 group, in comparison with DPSC-P1 group (Figure 2, *P *< 0.001). Western blot results further showed that the expression of osteoblast-specific matrix proteins (OPN, OSX, and BSP), odontoblast marker protein (dentin sialoprotein, DSP), and chondrocyte marker protein (COL II) was significantly elevated in DPSC-P9 cells, as compared with DPSC-P1 cells (Figure 3). There was no significant difference between two groups in the gene/protein expression (Figure 2, 3) for Runt-related transcription factor 2 (RUNX2), type I collagen (Col I), and osteonectin (ON). Since RUNX2 is an early osteoblastic marker [13], the similar levels of RUNX2 expression in DPSC-P1 and DPSC-P9 cells may imply that these two groups share almost the same percentage of immature osteoblasts.

### ALP and calcium deposition of STRO-1^+ ^DPSCs in the mineralization-inducing medium

**Figure 4:**
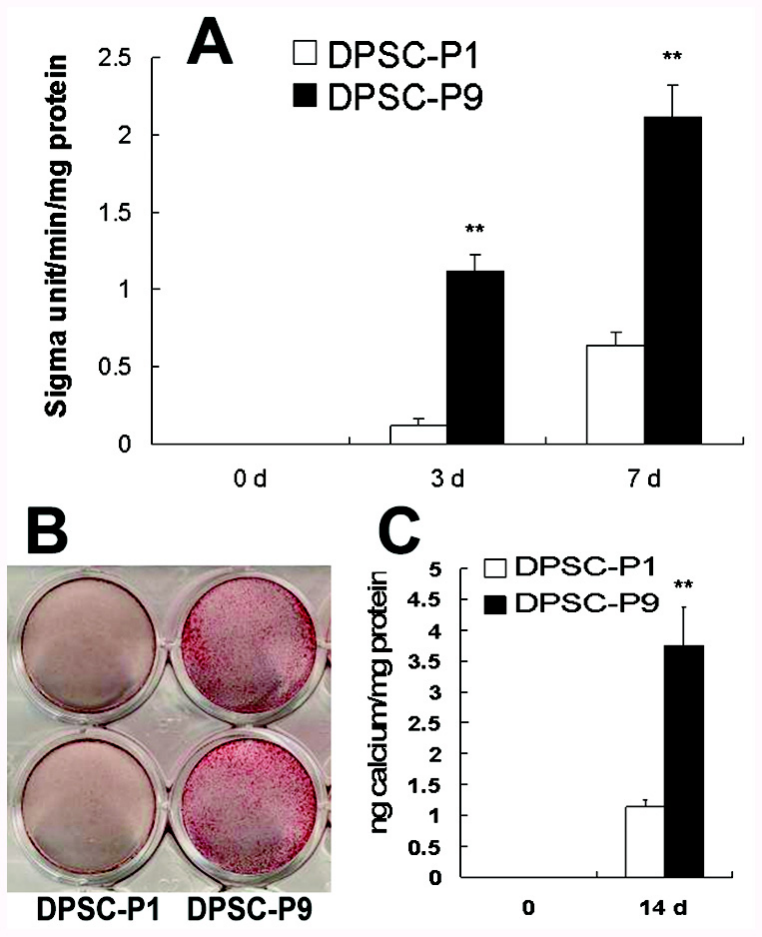
**ALP assay and alizarin red staining of DPSCs in the mineralization-inducing media**. (**A**) ALP concentrations in DPSCs at different passages. DPSC-P9 group presented a higher ALP level (*P *< 0.001) than DPSC-P1 group after 3 d or 7 d induction. (**B**) Alizarin red staining demonstrated that DPSC-P9 group generated more calcification nodules than DPSC-P1 group after 14 d induction in the mineralization-inducing media. (C) Calcium concentrations in DPSC-P9 group was significantly elevated (*P *< 0.001), as compared with DPSC-P1 group. Values are mean ± s.d., n = 9 (**P < 0.001, Student's t-test).

To further study their differentiation pace towards osteo-/odontoblast lineages *in vitro*, STRO-1^+ ^DPSCs at different passages were cocultured with the mineralization-inducing media. DPSC-P9 group in the mineralization-inducing media presented a higher ALP level than DPSC-P1 group at day 3 and day 7 respectively (Figure 4A, *P *< 0.001). The results of alizarin red staining demonstrated that more mineralization nodules were detected in DPSC-P9 group than in DPSC-P1 group after 14-day induction in the mineralization-inducing media (Figure 4B). The calcium concentrations in induced DPSC-P9 group were much higher than those in induced DPSC-P1 group (Figure 4C, *P *< 0.001).

### *In vivo *differentiation of STRO-1^+ ^DPSCs at the 1^st ^and 9^th ^passages

**Figure 5:**
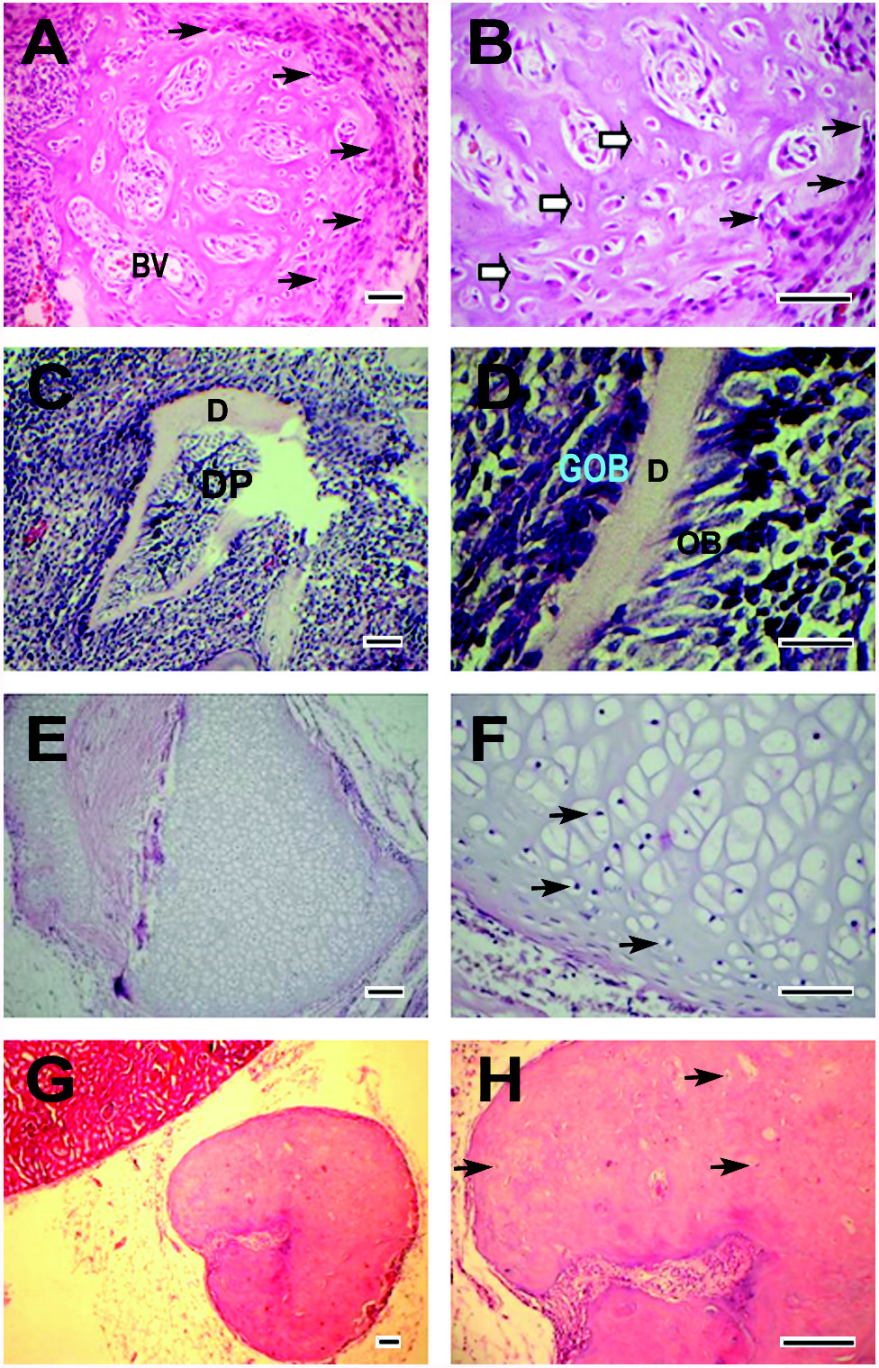
***In vivo *transplantation results of DPSC pellets at different passages (hematoxylin and eosin staining)**. (**A**) DPSC-P1 pellets generated bone tissues surrounded by osteoblasts (black arrows) at day 14, in which blood vessel-like structure (BV) can be observed. (**B**) A higher magnification of (**A**). Distinct osteocyte lacunae (open arrows) containing osteocytes were observed inside the bone structure and many osteoblasts (black arrows) located around the bone tissues. (**C**) 14-day DPSC-P1 pellets developed into a dentin-pulp complex containing dental pulp structures (DP). (**D**) A higher magnification of (**C**). Columnar odontoblasts (OB) arrayed orderly along the inner side of dentin matrix (D). Many globular odontoblasts (GOB) located along the outer side of dentin structure. (**E**) 14-day DPSC-P1 pellets formed cartilage tissues. (**F**) A higher magnification of (**E**). Many chondrocytes (black arrows) located inside the cartilage tissues. (**G**) All DPSC pellets at the 9^th ^passage bring about the formation of bone structures. No distinct osteoblast appeared around the mineralization tissue. (**H**) A higher magnification of (**G**). Osteocyte lacunae (black arrows) containing few osteocytes inside the bone tissues. Scale bars: 50 μm.

To further assess the self-differentiation ability of STRO-1^+ ^DPSCs *in vivo*, rat DPSC pellets at the 1^st ^and 9^th ^passages were transplanted into the renal capsules of adult rat hosts. All implants were retrieved at day 14 post-transplantation and processed for hematoxylin and eosin staining. *In vivo *transplantation results showed that all DPSC-P1 cell pellets gave birth to woven bone tissues (Figure 5A-B, 28/28), among which 9 samples (9/28) simultaneously produced dentin structures (Figure 5C-D) with typical odontoblasts aligning along the inner side of the predentin and 2 samples (2/28) generated cartilage structures (Figure 5E-F). Osteoblasts and osteocytes were distinct around or inside bone structures (Figure 5B), while chondrocytes can be found in the cartilage tissues (Figure 5F). All DPSC-P9 cell pellets (28/28, Figure 5G-H) *in vivo *developed into bone tissues with thicker matrix, less lacunae, and less osteocytes. No osteoblast can be observed around bone tissues in DPSC-P9 group (Figure 5G-H). There was a statistically significant difference (χ^2 ^= 10.72, *P *< 0.01) in the formation rate of dentin tissues between DPSC-P1 and DPSC-P9 pellets, whereas the differences between two groups were not statistically significant in the formation rate of cartilage (χ^2 ^= 2.07, *P *> 0.05) or bone (28/28 in both DPSC-P1 and DPSC-P9 groups) tissues.

## Discussion

Stem cell is a cell that can continuously give birth to unaltered daughters and also has the ability to generate progenitors that will differentiate into mature cell types. In this study, DPSC-P9 cells presented the enlarged cell bodies, decreased proliferation ability, upregulated marker gene/protein expression of different matrix-forming cells, as compared with DPSC-P1 cells. These findings indicate that STRO-1^+ ^DPSCs are spontaneously undergoing the multi-differentiation along different matrix-forming cell lineages with passage time. Many studies have proved that DPSCs can differentiate into odontoblast, osteoblast, and chondrocyte lineages in the presence of different inductive agents (e.g. growth factors, chemicals, stress, and high cell density culture) [14-17]. Our results revealed for the first time that DPSCs at the 9^th ^passage can spontaneously develop into odontoblasts, osteoblasts, and chondrocytes in the routine culture media, as indicated by the elevated gene/protein expression of *DSPP*/DSP, ALP, OPN, OSX, BSP, and COL II.

When transplanted into the renal capsules, DPSC-P1 cells can respectively generate bone, dentin, and cartilage structures after 14 days of *in vivo *incubation, further suggesting that these stem cells are composed of several interrelated subpopulations including the progenitors of odontoblasts, osteoblasts, and chondrocytes. The multi-differentiation capacity of these stem cells in different inductive media, as proven in many studies [10,14-17], may be attributed to these different subpopulations residing in DPSCs. When undifferentiated DPSCs are cultured in the inductive media (e.g. osteogenic medium), only one subpopulation (e.g. osteoblast progenitors) can grow in a prevalent way and give birth to the differentiated mature cells (e.g. osteoblasts).

STRO-1 is thought to be a putative cell surface marker for the isolation of stem cells from both human and rat dental pulps [18-20]. When recombined with the scaffold hydroxyapatite/tricalcium phosphate (HA/TCP), STRO-1^+ ^DPSCs can bring about the formation of abundant hard tissues in the subcutaneous area of nude mice [19]. The recombinations between STRO-1^+ ^DPSCs and HA/TCP *in vitro *can trigger the upregulation of several odontoblast-related gene expression including *Bsp*, *Dspp*, and dentin matrix protein 1 (*Dmp*1). In the present study, the most striking feature of STRO-1^+ ^DPSCs is their ability to self-differentiate into odontoblasts with passage time, as indicated by the expression of *DSPP *gene and DSP protein in DPSC-P9 cells. When transplanted *in vivo*, STRO-1^+ ^DPSCs at the 1^st ^passage can give rise to the formation of dentin-pulp-like complex containing typical columnar odontoblasts. These data strongly imply that *in situ *odontoblasts are derived from DPSCs in adult dental pulps. The terminal differentiation of odontoblasts in the adult stages thus may contain the following successive stages: DPSC, odontoblast precursor, preodontoblast, young odontoblast, mature (polarized) odontoblast, and aging odontoblast [21,22]. After the asymmetric cell division, DPSCs can exit from the self-renewal, narrow their differentiation potential to the odontoblast lineage and give rise to the differentiated daughter cells including odontoblast precursors, pre-odontoblasts, and terminally differentiated odontoblasts [21,22].

Although the marker genes/proteins of osteoblasts, odontoblasts, and chondrocytes were upregulated in STRO-1^+ ^DPSCs at the 9^th ^passage *in vitro*, these stem cells can only produce bone tissues *in vivo*, due to the following reasons. Firstly, DPSC-P9 cells presented a much stronger osteo-dentinogenic differentiation capacity than DPSC-P1 cells as indicated by elevated ALP levels and calcium deposition in the inductive medium. These differentiated stem cells at the 9^th ^passage have a stronger matrix-secreting ability, trap themselves in the fast-deposited matrix and ultimately result in the formation of a big mineralized mass with few cells inside. Secondly, to date, there is no optimal medium that can keep adult stem cells in an undifferentiated state during *in vitro *cell amplification. Long-term *in vitro *passage of stem cells may induce the cell differentiation, loss of self renewal ability [23], and decreased percentage of undifferentiated stem cells due to the asymmetric cell division. When these stem cells at the 9^th ^passage are implanted into the renal capsules, most stem cells are in a differentiated state, which conversely leads to the decrease of stem cell number, results in the loss of balance between stem cell proliferation and differentiation, and finally brings about the irregular tissue development [19]. Since the difference between dentin and bone is largely due to the different arrangement of matrix [24], irregular array of dentin and bone matrix secreted by odontoblasts and osteoblasts respectively may bring about the same appearance of bone-like calcification. Finally, the cell number of differentiated chondrocytes may be too limited (weak expression of COL II protein/gene) to perform the sustainable morphogenesis of cartilage in DPSC-P9 group. Together, DPSC-P9 cells *in vivo *can not perform the dentinogenesis or chondrogenesis but restrict their differentiation potential to the osteogenesis and generate the mineralized mass with few cells scattered inside.

## Conclusions

Our observations suggest that STRO-1^+ ^DPSCs consist of at least three interrelated subpopulations including the progenitors of odontoblasts, osteoblasts, and chondrocytes, which can form dentin, bone, and cartilage tissues respectively. The differentiation potential of these stem cells changes during serial cell passaging, and there is a restriction in the differentiation capacity of *in vivo *DPSCs at the 9^th ^passage, i.e., they can only differentiate along the osteoblast lineages. Further study is necessary to trace the mechanism of the restriction as well as the fate of other subpopulations residing in DPSCs.

## Methods

### Cell isolation and magnetic activated cell sorting (MACS)

All experiments were performed with the approval of Ethics Committee of Stomatological School of Nanjing Medical University (reference no. 200900128). All procedures were carried out according to the guidelines from Animal Care Committee of Nanjing Medical University. Multi-colony-derived DPSCs were enzymically isolated from dental pulps of human/rat molars as described before [8,9,25], and cultured in alpha-minimum essential medium (α-MEM; Gibco-BRL, Grand Island, NY) containing 10% fetal calf serum (FCS), 0.292 mg/mL glutamine, 100 units/mL penicillin G, 100 μg/mL streptomycin, 2.5 μg/mL ascorbic acid, and 25 mg/L bovine pituitary extract (Gibco-BRL). To obtain STRO-1^+ ^stem cells, DPSCs were indirectly sorted using immunomagnetic beads (Dynal Biotech, Oslo, Norway) according to the manufacturer's protocol. Briefly, approximately 5 × 10^6 ^cells were incubated with mouse anti-human STRO-1 supernatant (R&D systems, Minneapolis, MN) at 4°C for 30 minutes, washed with PBS/5% FCS, and resuspended with rat anti-mouse IgM-conjugated Dynabeads on a rotary mixer for 60 minutes. After washing, bead-positive cells were separated with a magnetic particle separator and subsequently placed into 75 cm^2 ^culture flasks (Costar, Cambridge, MA). Immunosorted DPSCs were cultured, passaged in the routine culture media (α-MEM) at 37°C in 5% CO_2_, and observed under the phase-contrast inverted microscope (Olympus). Approx 5% of DPSCs in the primary cells can be harvested by STRO-1-mediated MACS method. The biological features of STRO-1^+ ^DPSCs at the first (DPSC-P1) and ninth (DPSC-P9) passages were then investigated.

### Growth curve and population doubling time

Human STRO-1^+ ^DPSCs were respectively seeded into 96-well plates (Costar, Cambridge, MA) at a density of 1 × 10^3 ^cells/well. For eleven consecutive days, cell counting was performed daily with Coulter Counter (Beckman Coulter, Fullerton, CA). The average number of cells was plotted on a graph and population doubling time (PDT) was calculated according to Patterson formulation [26].

### Realtime reverse transcriptase-polymerase chain reaction (realtime RT-PCR)

Total cellular RNA was isolated by adding Trizol reagent (Invitrogen, Carlsbad, CA) to human DPSC samples. First-strand cDNA was synthesized using SuperScript^® ^III cDNA Synthesis Kit (Invitrogen). Real-time RT-PCR was performed using the QuantiTect SYBR Green PCR kit (QiaGen) and Icycler iQ Multi-color real-time PCR detection system. Primers used were: *ALP*, 5'-TGATGAATGCTTGCGAAGGGT-3' (forward) and 5'-TCTCCGCATTGCATTTTCTGCT-3' (reverse); *BSP*, 5'-TCTGTTTTGCTGATGGGCTTGG-3' (forward) and 5'-AAAAGGTCTTGATGGCCCCTTG-3' (reverse); *DSPP*, 5'-ATATTGAGGGCTGGAATGGGGA-3' (forward) and 5'-TTTGTGGCTCCAGCATTGTCA-3' (reverse); *COL *I, 5'-TCTCCATCCTTGCCGTTGATTG-3' (forward) and 5'-TCCCCACCTTCAAAATTCTGGG-3' (reverse); *COL *II, 5'-TTGGGTTTGCAACGGATTGTG-3' (forward) and 5'-AGCAGGAATTCGGTGTGGACAT-3' (reverse); *ON*, 5'-AACAAGCATGTAAGGGCCCGAT-3' (forward) and 5'-TCCAGAGCATTTTCATCCAGGG-3' (reverse); *OPN*, 5'-CATTGCAGGTCTCCTGGAACAA-3' (forward) and 5'-TTAGCATCGGTGGTTTCCGTTC-3' (reverse); *RUNX*2, 5'-TGGAACATCTCCATCAAGGCAG-3' (forward) and 5'-TCAGGATATTCGGGACGTTGGA-3' (reverse); OSX, 5'-TGGAAAGCCAGTCTCATGGTGA-3' (forward) and 5'-TTGGGTATCTCCTTGCATGCCT-3' (reverse); *GAPDH*, 5'-CGGCTACCACATCCAAGGAA-3' (forward) and 5'-AGCCACATCGCTCAGACACC-3' (reverse).

### Western blot analysis

Human STRO-1^+ ^DPSCs were collected and lysed in RIPA buffer (10 mM Tris-HCL, 1 mM EDTA, 1% sodium dodecyl sulfate (SDS), 1% Nonidet P-40, 1: 100 proteinase inhibitor cocktail, 50 mM β-glycerophosphate, 50 mM sodium fluoride). The detergent-soluble fractions were subjected to 7.5% SDS-PAGE gels according to the standard protocol and transferred to PVDF membrane by a semi-dry transfer apparatus (Bio-Rad). After blocking in 5% (w/v) skim milk diluted in Tris-buffered saline (TBS) buffer (blocking buffer, 50 mM Tris-HCL, and 150 mM NaCL) at room temperature for 2 h, the membranes were incubated with primary antibodies overnight at 4°C. Primary antibodies included: polyclonal antibody against dentin sialoprotein (DSP; 1:500; Santa Cruz), monoclonal antibody against ON (1:100; Novocastra), monoclonal antibody against COL I (1:2,000; Abcam), monoclonal antibody against BSP (1:1,000; Abcam), polyclonal antibody against OPN (1:500; Santa Cruz), and polyclonal antibody against COL II (1:1,000; Abcam), polyclonal antibody against RUNX2 (1:500; Santa Cruz), polyclonal antibody against OSX (1:1,000; Abcam), monoclonal antibody against α-tubulin (1:100,000; Sigma-Aldrich). The membranes were then washed with TBST (0.2% Tween-20 in blocking buffer), incubated with horseradish peroxidase-conjugated anti-rabbit or anti-mouse IgG (Promega), visualized by SuperSignal reagents (Pierce) and exposed to Kodak X-ray films.

### ALP assay and alizarin red staining

Human STRO-1^+ ^DPSCs at different passages were cultured using 12-well cell culture plates in the mineralization-inducing medium containing 100 μM ascorbic acid, 2 mM β-glycerophosphate, and 10 nM dexamethasone. Then, ALP activity and calcium deposition were evaluated at different time point. ALP assay was performed with an ALP kit according to the manufacturer's instructions (Sigma-Aldrich) and normalized on the basis of protein concentrations. To assess the mineralization, cells were induced for 14 days, fixed with 70% ethanol and stained with 2% alizarin red (Sigma-Aldrich). Calcium content in mineralized nodules formed by DPSCs at different passages was quantitatively determined according to our previous protocols [27]. Briefly, alizarin red was destained with 10% cetylpyridinium chloride (CPC) in 10 mM sodium phosphate for 60 min at room temperature. Calcium concentrations were determined by the absorbance measurement on a multiplate reader at 562 nm using a standard calcium curve. The protein content was quantitatively determined using Bio-Rad protein assay solution (Bio-Rad Laboratories, CA) after the removal of CPC solution from the samples. The final calcium concentrations were normalized with the total protein content prepared from the duplicate plates.

### Cell transplantation

28 renal capsules from 14 adult Sprague-Dawley (SD) rats were used for the allogenic transplantation. STRO-1^+ ^DPSC pellets at the 1^st ^or 9^th ^passages were harvested from rat dental pulps, maintained in α-MEM supplemented with 20% FCS, and incubated in tubes for 3 h in order to make them well aggregated. Then, DPSC pellets were statically loaded onto the absorbable gelatin sponges (AGS; Nanjing Pharmaceuticals Inc., China), which act as a carrier to facilitate the transfer of cell pellets. Cell pellets on the AGS were then seeded directly into the renal capsules of rat hosts with modified pipette tips. Each renal capsule harbored two cell pellets containing 1 × 10^5 ^cells. The resulting tissues (28 implants in each group) were recovered at day 14 post-transplantation, fixed in 4% polyoxymethylene, and processed for hematoxylin and eosin staining (H&E).

### Statistical analyses

Statistical analyses of the data were performed by student's *t *test and Chi-square test with SPSS v 12.0 software (SPSS, Chicago, IL, USA). Differences with p-values < 0.05 were considered significant.

## Authors' contributions

JY performed the study design, cell isolation, cell culture, realtime RT-PCR, data collection and manuscript writing. HH carried out western blot and growth kinetics. CT undertaken ALP assay and alizarin red staining. GZ fulfilled the transplantation experiments, H&E staining, and sample analyses. YL participated in the cell sorting and western blot. RW contributed to the sample design, statistical analysis and manuscript improvement. JS helped to design the study and provided comments on the manuscript. YJ gave feedback and guidance on the hypotheses, and helped to draft the manuscript. All authors read and approved the final manuscript.
